# Antimicrobial susceptibility of Enterobacterales causing infection in the elderly: focus on aztreonam-avibactam and recently approved β-lactamase inhibitor combinations

**DOI:** 10.1093/jacamr/dlaf189

**Published:** 2025-10-22

**Authors:** Helio S Sader, Rodrigo E Mendes, John H Kimbrough, Krisztina M Papp-Wallace, Marisa L Winkler, Mariana Castanheira

**Affiliations:** Element Iowa City (JMI Laboratories), North Liberty, IA, USA; Element Iowa City (JMI Laboratories), North Liberty, IA, USA; Element Iowa City (JMI Laboratories), North Liberty, IA, USA; Element Iowa City (JMI Laboratories), North Liberty, IA, USA; Element Iowa City (JMI Laboratories), North Liberty, IA, USA; Element Iowa City (JMI Laboratories), North Liberty, IA, USA

## Abstract

**Background:**

The US elderly population (≥65 years old) increased markedly in the last decades, and infections are responsible for approximately one-third of all deaths in this population. We evaluated the antimicrobial susceptibility of Enterobacterales causing infection in elderly patients in US hospitals.

**Methods:**

Unique patient clinical isolates were consecutively collected from 72 US hospitals in 2021–2023 and tested for susceptibility by broth microdilution. Results for 10 574 Enterobacterales from elderly patients were analysed and compared with 9793 isolates from adult patients (18–64 years old). Carbapenem-resistant Enterobacterales (CRE) were screened for carbapenemases by whole-genome sequencing.

**Results:**

All isolates from elderly patients were inhibited at aztreonam-avibactam MIC of ≤8 mg/L (>99.9% susceptible at ≤4 mg/L). Ceftazidime-avibactam and meropenem-vaborbactam were very active against Enterobacterales overall (≥99.7% susceptible) but exhibited limited activity against CRE (70.4%–71.6% susceptible). The most active agents against CRE were aztreonam-avibactam (98.8% susceptible), cefiderocol (96.3% susceptible) and tigecycline (96.3% susceptible). Susceptibility rates of isolates from the elderly were comparable (±≤ 2.6%) with those from the adult population; however, the frequencies of CRE and MDR phenotypes were lower among the elderly than adults. The most common carbapenemase among CREs from elderly patients were *Klebsiella pneumoniae* carbapenemases (55.6% of CRE) and NDM (24.7%); a metallo-β-lactamase was identified in 28.4% of CRE isolates.

**Conclusions:**

Enterobacterales causing infections in the elderly population showed a similar antimicrobial resistance profile but a lower frequency of CRE and MDR isolates to those causing infection in the adults.

## Introduction

The US elderly population (≥65 years old) increased in the decade from 2010 to 2020 at the fastest rate since the first decade measured (1880 to 1890). The elderly population rate increased almost five times faster than the overall population in the last 100 years (1920 to 2020), reaching 55.8 million or 16.8% of the US population in 2020 (https://www.census.gov/library/stories/2023/05/2020-census-united-states-older-population-grew.html). Moreover, the frequency of hospital-associated bacterial infections has increased with age, not only because of an increased hospitalization rate, but also due to an increased risk of infection per day of hospitalization.^1,2^

Infections account for approximately one-third of all deaths in the elderly, and this population has a higher risk of exposure to antimicrobial-resistant bacteria. Some types of infection are more common in the elderly than in adults (18 to 64 years old), particularly bacterial infections, such as urinary tract infection (UTI), pneumonia, bloodstream infections (BSIs), endocarditis, and skin and soft tissue infection (particularly diabetic foot infection).^2^ Infections in the elderly are not simply more common and more severe, but they also have particular characteristics with respect to clinical presentation, microbial epidemiology, laboratory results and treatment.^3^ Notably, early detection is challenging in the elderly since the typical signs and symptoms, such as fever and leucocytosis, may be absent.^4^

The epidemiology of infection is also different in the elderly population compared with adults, but data in the literature on this phenomenon are scarce. The changes in microbiology may be related to age itself and/or to comorbidities. Published data regarding the microbial epidemiology of specific infections as well as the antimicrobial susceptibility are valuable; however, local data should be obtained to improve patient management.^2,3^

Multidrug-resistant (MDR) Gram-negative bacteria are leading causes of hospital-associated infections, including those affecting the elderly. Remarkably, carbapenem-resistant Enterobacterales (CRE) has increased in the last decade, and carbapenem resistance among Enterobacterales is generally due to the production of carbapenemases that are able to hydrolyse most β-lactams currently available for clinical use.^5^ Various antimicrobial agents targeting CRE were licensed by the US FDA and the EMA in the last few years, including ceftazidime-avibactam, meropenem-vaborbactam, imipenem-relebactam and cefiderocol.^6^ The approval of these agents represented a valuable advance in the treatment of infections caused by MDR Gram-negatives, specially CRE; however, increasing resistance to these agents is being observed in some US hospitals.^7^

Aztreonam-avibactam was recently approved by the EMA in the European Union for treatment of adults with complicated intra-abdominal infection (IAI), complicated UTI (cUTI), hospital-acquired pneumonia, including ventilator-associated pneumonia, and infections due to aerobic Gram-negative bacteria in adults with limited treatment options (https://www.ema.europa.eu/en/news/new-antibiotic-fight-infections-caused-multidrug-resistant-bacteria; accessed on 4 June 2025) and by the US FDA for treatment of complicated IAI.^8^ Aztreonam is stable to hydrolysis by metallo-β-lactamases (MBLs) and avibactam protects aztreonam from hydrolysis by clinically relevant serine β-lactamases, such as extended-spectrum β-lactamase (ESBL), chromosomal derepressed AmpC and *Klebsiella pneumoniae* carbapenemases (KPC). We evaluated the antimicrobial susceptibility of Enterobacterales causing infection in elderly patients in US hospitals.

## Methods

### Organism collection

The isolates were collected from 72 US medical centres in 2021–2023 as part of the International Network for Optimal Resistance Monitoring (INFORM) programme.^9^ Each participating centre was requested to provide a certain number of consecutive patient unique isolates from designated infection types collected during a specific period of the year, independent of patient age. In this investigation, we assessed the susceptibility results of all Enterobacterales isolates from elderly patients (≥65 years old; *n* = 10 574) and compared with all Enterobacterales isolates from adults (18–64 years old; *n* = 9793) collected in 2021–2023.

### Susceptibility testing

Isolates were susceptibility tested by the reference broth microdilution method as described by the CLSI.^10^ Aztreonam-avibactam, ceftazidime-avibactam, imipenem-relebactam, ceftolozane-tazobactam and piperacillin-tazobactam were tested with a β-lactamase inhibitor at a fixed concentration of 4 mg/L; meropenem-vaborbactam was tested with vaborbactam at a fixed concentration of 8 mg/L.^10,11^ Cefiderocol was tested in iron-depleted media.^10^ Owing to the increased cost of testing cefiderocol in iron-depleted media and the reduced availability of cefiderocol powder during the investigation, cefiderocol was only tested against CRE isolates. MIC values were interpreted according to CLSI and/or US FDA breakpoint criteria unless noted. Isolates were categorized as MDR according to criteria defined in 2012 by the joint European and US Centers for Disease Control.^12^ These criteria define MDR as non-susceptible to ≥1 agent in ≥3 antimicrobial classes and XDR as susceptible to ≤2 classes. The ESBL phenotype was defined for *Escherichia coli*, *Klebsiella oxytoca*, *K. pneumoniae* and *Proteus mirabilis* as an MIC value ≥2 mg/L for ceftriaxone, ceftazidime and/or aztreonam.^11^ CRE isolates were defined as displaying imipenem or meropenem MIC values at ≥4 mg/L. Imipenem was not applied to *Proteus mirabilis* or indole-positive Proteeae due to their intrinsically elevated MIC values. Categorical interpretations followed CLSI^11^ and/or US FDA criteria (https://www.fda.gov/drugs/development-resources/antibacterial-susceptibility-test-interpretive-criteria), unless otherwise noted. Results were stratified by age group, infection type and resistant subsets.

### Statistical analysis

Statistical analysis was performed using Epi Info^TM^ 7 (Centers for Disease Control, Atlanta, GA, USA).

### Screening for β-lactamases

CRE isolates were screened for β-lactamase–encoding genes by whole-genome sequencing using next-generation sequencing. Total genomic DNA was prepared using the KingFisher Cell and Tissue DNA kit (ThermoFisher Scientific, Waltham, MA, USA) or the MagMax DNA Multi-Sample Ultra 2.0 extraction kit (ThermoFisher) on a KingFisher Flex Magnetic Particle Processor (ThermoFisher). DNA libraries were constructed using either the Nextera XT library construction protocol and index kit or the Illumina DNA prep (Illumina, San Diego, CA, USA). Sequencing performed on either a NextSeq 1000 Sequencer using NextSeq1000/2000 P2 Reagents (300 cycles) or a MiSeq Sequencer with a MiSeq Reagent Kit v.3 (600 cycles). The generated FASTQ files were assembled using SPAdes Assembler and subjected to proprietary software (Element Iowa City, JMI Laboratories) for screening of β-lactamase genes.^13^

## Results

**Figure 1.**
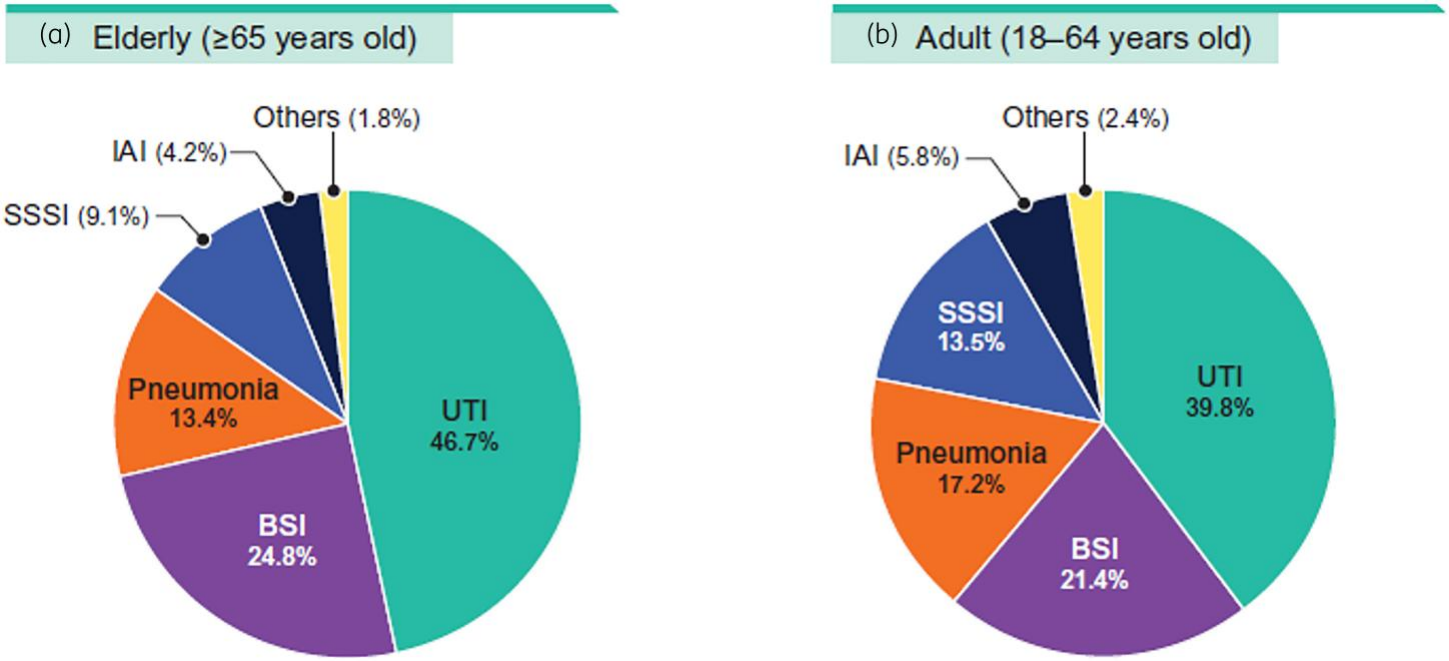
Distribution of isolates by infection sources: (a) elderly and (b) adult. SSSI, skin and skin structure infection.

Enterobacterales isolates from both elderly and adult patients were mainly from UTI (39.8%–46.7%), BSI (21.4%–24.8%) and pneumonia (13.4%–17.2%; Figure 1). Aztreonam-avibactam (MIC_50/90_,  ≤ 0.03/0.12 mg/L; 99.9% susceptible at ≤4 mg/L), ceftazidime-avibactam (MIC_50/90_, 0.12/0.25 mg/L; 99.7% susceptible), meropenem-vaborbactam (MIC_50/90_, 0.03/0.06 mg/L; 99.8% susceptible) and meropenem (MIC_50/90_, 0.03/0.06 mg/L; 99.2% susceptible) were highly active against Enterobacterales from elderly patients with >99% susceptibility; whereas imipenem-relebactam (MIC_50/90_, 0.12/1 mg/L; 92.8% susceptible) and ceftolozane-tazobactam (MIC_50/90_, 0.25/1 mg/L; 94.5% susceptible) were slightly less active than those compounds (Tables 1 and 2). Among non–β-lactam compounds, the most active agents against isolates from elderly patients were tigecycline (MIC_50/90_, 0.25/2 mg/L; 96.0% susceptible) and amikacin (MIC_50/90_, 2/4 mg/L; 95.3% susceptible; Table 1). Notably, isolates from elderly patients exhibited susceptibility rates comparable to those from isolates from adults for most antimicrobial agents. Differences of susceptibility rates between elderly patients and adults were 1.2% or less except for trimethoprim-sulfamethoxazole (2.6%; Table 1).

**Table 1.** Antimicrobial susceptibility of Enterobacterales from elderly and adult patients

Antimicrobial agent (no. of isolates)	Elderly		Adult	
	%S^a^	%R^a^	%S^a^	%R^a^
Enterobacterales (10 574/9793)				
Aztreonam-avibactam	>99.9	0.0	99.9	0.1
Ceftazidime-avibactam	99.7	0.3	99.6	0.4
Meropenem-vaborbactam	99.8	0.2	99.6	0.3
Imipenem-relebactam^b^	92.8	1.5	93.3	1.6
Ceftolozane-tazobactam	94.5	4.0	93.7	4.8
Piperacillin-tazobactam	88.8	7.7	87.7	9.0
Ceftriaxone	81.3	17.8	80.9	18.1
Ceftazidime	85.7	12.7	85.3	13.0
Cefepime	88.8	8.3	88.1	8.9
Aztreonam	85.4	13.1	84.6	13.8
Meropenem	99.2	0.6	98.5	1.3
Imipenem	90.4	2.0	90.5	2.6
Levofloxacin	81.4	16.0	82.6	14.6
Gentamicin	92.2	7.2	91.2	8.1
Amikacin	95.3	0.9	95.6	1.1
TMP-SMX^c^	81.7	18.3	79.1	20.9
Tigecycline	96.0	0.3	95.6	0.4
Colistin^e^		21.0		19.9
CRE (81/141)				
Aztreonam-avibactam	98.8	0.0	97.9	1.4
Ceftazidime-avibactam	71.6	28.4	75.2	24.8
Meropenem-vaborbactam	70.4	25.9	70.9	24.1
Imipenem-relebactam^b^	65.4	30.9	66.0	31.2
Cefiderocol^d^	96.3	3.7	92.9	5.0
Levofloxacin	32.1	56.8	31.9	56.0
Gentamicin	67.9	27.2	63.1	31.9
Amikacin	63.0	22.2	66.7	24.1
TMP-SMX^c^	43.2	56.8	40.4	59.6
Tigecycline	96.3	1.2	95.0	0.0
Colistin^e^		16.0		17.0

^a^Criteria as published by CLSI,^11^ except for aztreonam-avibactam and tigecycline where US FDA breakpoints were applied.

^b^All Enterobacterales species were included in the analysis, but CLSI excludes organisms from the family Morganellaceae.

^c^Trimethoprim-sulfamethoxazole.

^d^Cefiderocol was only tested against CRE isolates. Susceptible at ≤4 mg/L, intermediate at 8 mg/L and resistant at ≥16 mg/L.^11^

^e^CLSI does not publish a susceptible breakpoint for colistin, only intermediate (≤2 mg/L) or resistant (≥4 mg/L).^11^

**Table 2.** Activity of β-lactamase inhibitor combinations against Enterobacterales from elderly patients

Organism/resistant	Percentage susceptible (MIC_90_ in mg/L)					
subset/infection (no.)	ATM-AVI	CAZ-AVI	MEM-VAB	IMI-REL	TOL-TAZ	PIP-TAZ
Enterobacterales (10 574)	>99.9 (0.12)	99.7 (0.25)	99.8 (0.06)	92.8 (1)	94.5 (1)	88.8 (16)
CRE (81)	98.8 (0.5)	71.6 (>32)	70.4 (>32)	65.4 (>8)	6.2 (>16)	4.9 (>128)
ESBL phenotype (1297)	99.9 (0.12)	98.7 (0.5)	98.8 (0.06)	95.6 (0.25)	85.9 (4)	68.3 (>128)
MDR phenotype (2089)	99.9 (0.5)	98.7 (1)	98.9 (0.06)	97.0 (0.25)	72.5 (16)	51.9 (>128)
UTI (4935)	>99.9 (0.12)	99.8 (0.25)	99.8 (0.06)	92.0 (1)	95.8 (1)	91.4 (8)
BSI (2620)	100.0 (0.12)	99.7 (0.25)	99.7 (0.06)	94.8 (0.5)	96.1 (1)	90.8 (8)
Pneumonia (1415)	100.0 (0.25)	99.3 (0.5)	99.6 (0.06)	94.7 (1)	88.7 (4)	77.6 (64)
SSSI (965)	100.0 (0.12)	99.9 (0.25)	99.8 (0.06)	86.2 (2)	93.8 (1)	86.8 (16)
IAI (446)	99.8 (0.12)	99.8 (0.25)	99.8 (0.06)	94.3 (0.5)	91.3 (2)	86.5 (32)
Other infections (193)	100.0 (0.12)	100.0 (0.25)	100.0 (0.06)	93.5 (1)	92.7 (1)	89.1 (16)

ATM-AVI, aztreonam-avibactam; CAZ-AVI, ceftazidime-avibactam; MEM-VAB, meropenem-vaborbactam; IMI-REL, imipenem-relebactam; TOL-TAZ, ceftolozane-tazobactam; PIP-TAZ, piperacillin-tazobactam; SSSI, skin and skin structure infection.

The most active agents against CRE isolates from elderly patients (*n* = 81; 0.8%) were aztreonam-avibactam (MIC_50/90_, 0.25/0.5 mg/L; 98.8% susceptible), cefiderocol (MIC_50/90_, 1/4 mg/L; 96.3% susceptible) and tigecycline (MIC_50/90_, 0.5/2 mg/L; 96.3% susceptible; Table 1). Remarkably, ceftazidime-avibactam (MIC_50/90_, 2/>32 mg/L; 71.6% susceptible), meropenem-vaborbactam (MIC_50/90_, 0.25/>32 mg/L; 70.4% susceptible) and imipenem-relebactam (MIC_50/90_, 0.25/>8 mg/L; 65.4% susceptible) exhibited limited activity against these organisms. Again, CRE isolates from adults showed susceptibility rates similar to CRE isolates from elderly patients for most antimicrobial agents (Table 1).

**Figure 2.**
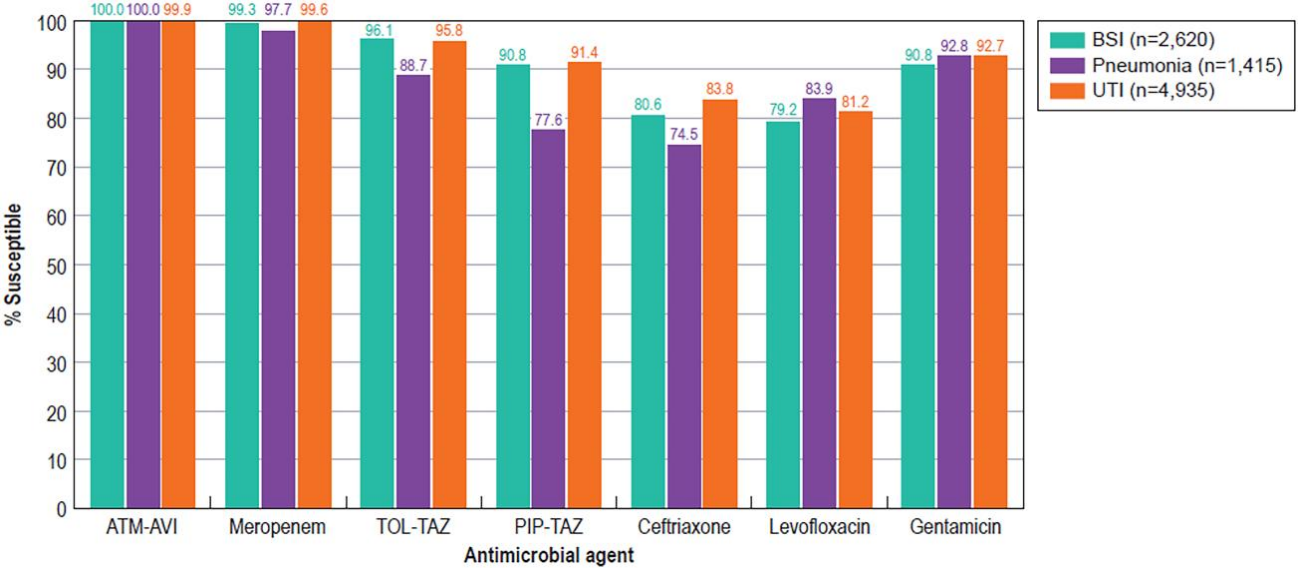
Activity of selected agents against elderly isolates stratified by infection source. ATM-AVI, aztreonam-avibactam; TOL-TAZ, ceftolozane-tazobactam; PIP-TAZ, piperacillin-tazobactam.

Table 2 summarizes the activities of the β-lactamase inhibitor combinations against Enterobacterales-resistant subsets as well as the isolates stratified by infection type. Aztreonam-avibactam was active against ≥99.8% of isolates (MIC_90_, 0.12–0.5 mg/L) from all subsets except CRE (98.8% susceptible). Only two isolates (0.02%) were not susceptible to aztreonam-avibactam, both with an MIC of 8 mg/l (intermediate per US FDA criteria). Ceftazidime-avibactam (≥98.7% susceptible and MIC_90_ of 0.25–1 mg/L for all subsets except CRE) and meropenem-vaborbactam (≥98.8% susceptible and MIC_90_ of 0.06 mg/L for all subsets except CRE) were highly active against all subsets except CRE (Table 2). Notably, isolates from patients with pneumonia showed lower susceptibility rates for ceftolozane-tazobactam (88.7% versus 91.4%–96.1%) and piperacillin-tazobactam (77.6% versus 86.5%–91.4%) when compared with isolates from other infection types (Table 2). Moreover, when compared with isolates from patients with BSI and UTI, isolates from patients with pneumonia also showed lower susceptibility to meropenem and ceftriaxone, but higher susceptibility to levofloxacin (Figure 2).

**Figure 3.**
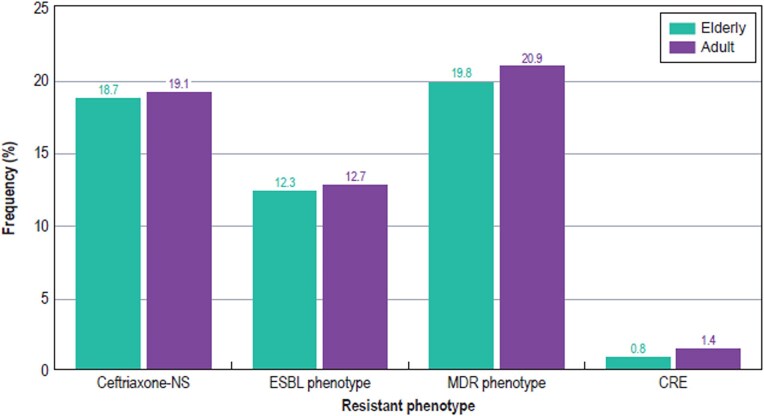
Frequency of selected resistant phenotypes. NS, non-susceptible.

The frequencies of selected resistance phenotypes among isolates from elderly patients and adults are shown in Figure 3. Notably, the occurrence of CRE was markedly lower among isolates from elderly patients compared with isolates from adults (0.8% versus 1.4%; *P* < 0.001; OR: 0.402–0.695). Moreover, the occurrences of ceftriaxone-non-susceptible Enterobacterales (18.7% versus 19.1%; *P* = 0.44; OR: 0.907–1.044), ESBL phenotype (12.3% versus 12.7%; *P* = 0.48; OR: 0.893–1.055) and MDR (19.8% versus 20.9%; *P* = 0.04; OR: 0.894–0.996) were slightly lower among isolates from elderly patients compared with isolates from adults (Figure 3).

**Figure 4.**
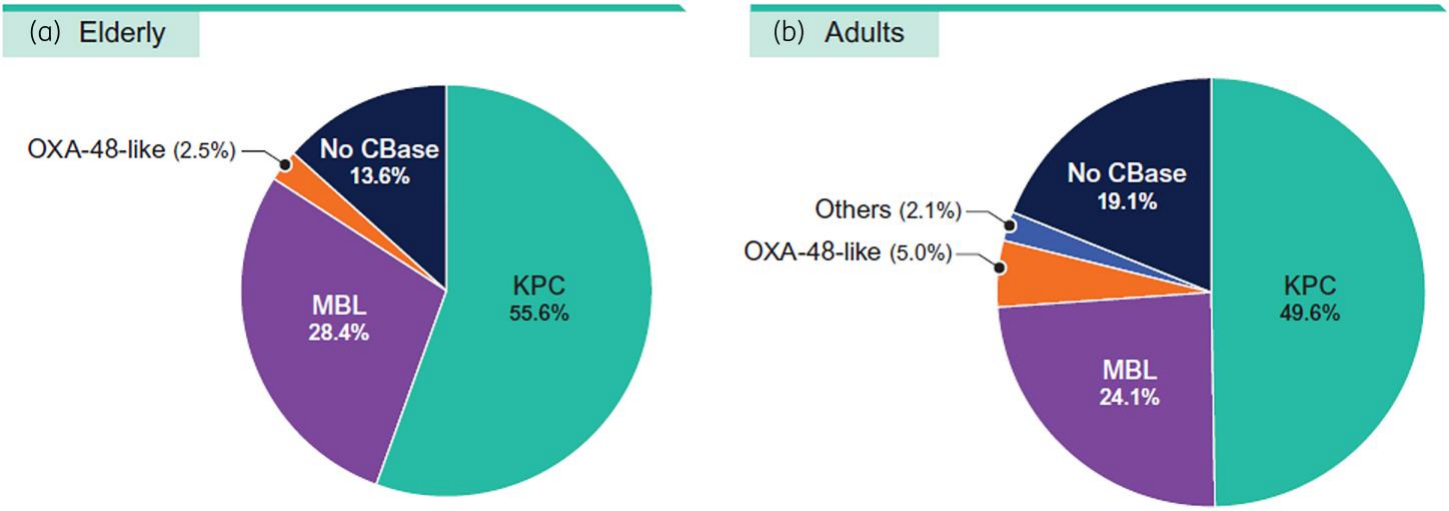
Frequencies of carbapenemases (CBase) among CRE isolates: (a) elderly and (b) adult. One isolate from elderly and four isolates from adults produced an MBL and a KPC and these isolates were included in the respective MBL groups. Moreover, one isolate from elderly and one isolate from adults produced an MBL and an OXA-48-like and these isolates were also included in the respective MBL groups.

**Figure 5.**
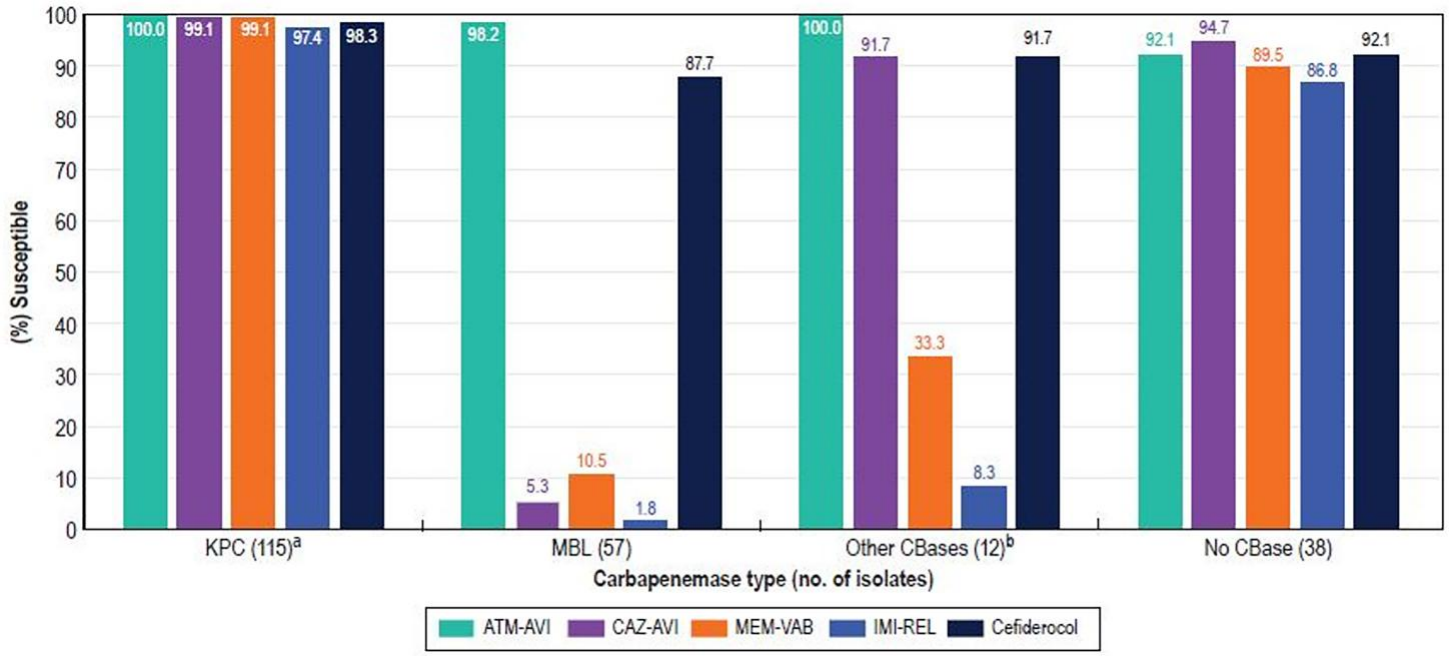
Antimicrobial activity of newer β-lactamase inhibitor combinations and cefiderocol against CRE from both elderly and adults combined and stratified by β-lactamase type. (a) Exclude isolates co-producing metallo-beta-lactamase. (b) Includes OXA-48 type (9), SME-2 (2), and IMI-4 (1). CBase, carbapenemase; ATM-AVI, aztreonam-avibactam; CAZ-AVI, ceftazidime-avibactam; MEM-VAB, meropenem-vaborbactam; IMI-REL, imipenem-relebactam.

A carbapenemase was identified in 86.4% (70/81) of CRE isolates from elderly patients and 80.9% (114/141) of CRE isolates from adults (Figure 4). The most common carbapenemase observed among CRE from elderly patients was KPC type (55.6% of CREs) and NDM type (24.7%). KPC (49.6%) and NDM (21.3%) types were also the most common carbapenemases detected among CRE from adult patients. Notably, 28.4% of CRE from elderly patients and 24.1% of CRE from adults carried an MBL, which confers resistance to ceftazidime-avibactam, meropenem-vaborbactam and imipenem-relebactam. Thus, the activities of the newer β-lactamase inhibitor combinations and cefiderocol against CRE isolates varied markedly according to the type of carbapenemase produced by the organism (Figure 5). All five compounds (aztreonam-avibactam, ceftazidime-avibactam, meropenem-vaborbactam, imipenem-relebactam and cefiderocol) were highly active (≥97.4% susceptibility) against KPC producers and showed 86.8% to 94.7% activity against CRE isolates that did not produce a carbapenemase. By contrast, only aztreonam-avibactam (98.2% susceptible) and cefiderocol (87.7% susceptible) were active against MBL producers, and only aztreonam-avibactam (100.0% susceptible), ceftazidime-avibactam (91.7% susceptible) and cefiderocol (91.7% susceptible) showed good activity against the group of CRE isolates that produced OXA-48–like, SME-2 or IMI-4 (Figure 5). Among NDM producers (*n* = 50), 98.0% of isolates were susceptible to aztreonam-avibactam and 86.0% of isolates were susceptible to cefiderocol. Cefiderocol non-susceptible NDM producers included NDM-1 (4 isolates) and NDM-5 (3 isolates) producers. Moreover, 11 NDM producers (22.0%) exhibited a cefiderocol MIC of 4 mg/L (data not shown in Tables).

## Discussion

A few studies evaluating the effect of ageing on the incidence of infection and on its microbiological characteristics and mortality have been reported from countries where life expectancy increased.^14–16^ However, susceptibility data on bacteria causing infection in the elderly population is still scarce.

Da Silva *et al*. evaluated the impact of ageing on the occurrence of BSI and on the microbiological profile of these infections, with a focus on MDR bacteria and on mortality resulting from BSI, in a tertiary public university hospital in Brazil. Their results indicated that ageing plays an important role in the increase in morbidities and mortality resulting from BSI in Brazil, but age was not significantly associated with MDR-related BSI.^14^

Gravey *et al*. assessed the bacteria isolated from urine specimen of elderly patients and their antimicrobial susceptibility in a 3-year retrospective study performed in a French university hospital.^17^ Results obtained with the elderly population (≥65 years old) were compared with those of adults (18 to 64 years old). The investigators found a higher proportion of Gram-negative bacteria and a higher frequency of ESBL-producing Enterobacterales in elderly patients compared with adults.^17^

The present study evaluated >10 000 Enterobacterales causing infection in elderly patients from 72 US hospitals and compared the results with a similar number of Enterobacterales isolates causing infection in the adult population in the same hospitals at the same time period. Importantly, we evaluated the activity of recently approved β-lactams active against MDR Gram-negatives. In general, susceptibility rates of isolates from elderly patients were comparable (±≤ 2.6%) to those from the adult population; however, the frequency of CRE, defined as resistance (MIC ≥4 mg/L) to imipenem or meropenem, was significantly lower among isolates from the elderly population than the adults (0.8% versus 1.4%; *P* < 0.001). The frequency of MDR organisms (resistance to >3 classes) was also lower among the elderly population (19.8% versus 20.9%; *P* = 0.04). Moreover, contrary to Gravey *et al*.,^17^ we found a slightly lower frequency of isolates showing an ESBL phenotype among elderly patients compared with adults (12.3% versus 12.7%; *P* = 0.48).

Possible reasons for finding higher frequencies of CRE and MDR among adults compared with the elderly in our studies includes higher frequency of patients with pneumonia (17.2% versus 13.4%; Figure 1), with healthcare-associated infections (17.6% versus 13.2%), and in ICU (18.2% versus 14.6%) among adults compared with the elderly. However, evaluation of other epidemiologic factors, such as previous use of antibiotics and length of hospitalization prior to infection among others, would be necessary to better understand these findings.

Our results showed that the newer β-lactamase inhibitor combinations (BLICs), aztreonam-avibactam, ceftazidime-avibactam and meropenem-vaborbactam, exhibited almost complete coverage (99.6%–> 99.9% susceptibility) against Enterobacterales from elderly patients and adults. Meropenem was also highly active against isolates from both groups (98.5%–99.2% susceptibility). Notably, susceptibility rates were moderately low for ceftriaxone (80.9%–81.3% susceptible), ceftazidime (85.3%–85.7% susceptible) and levofloxacin (81.4%–82.6% susceptible) in both age groups.

It is also important to note that, although the frequencies of CRE were relatively low in both age groups, CRE isolates demonstrated high resistance rates to most antimicrobial agents, including ceftazidime-avibactam (24.8%–28.4% resistant), meropenem-vaborbactam (24.1%–25.9% resistant) and imipenem-relebactam (30.9%–31.2% resistant). Elevated resistance to these BLICs has been related to the increased frequency of MBL producers among CRE in some US medical centres.^7,9,18,19^ As has been reported by our group as well as other investigators, the only antimicrobial agents active against MBL-producing CRE isolates were aztreonam-avibactam (98.2% susceptible), cefiderocol (87.7% susceptible) and tigecycline (93.0% susceptible).^6,9,20,21^

In conclusion, Enterobacterales causing infections in the elderly population showed similar antimicrobial resistance profiles but a lower frequency of CRE and MDR isolates to those causing infection in the adults. Aztreonam-avibactam demonstrated almost complete activity against Enterobacterales causing infection in both age groups and retained potent activity against CRE, including MBL producers and isolates with ESBL or MDR phenotypes. The frequency of MBL-producing Enterobacterales appears to be increasing rapidly and represented a significant proportion of CRE isolates from elderly (28.4%) and adult (24.1%) patients.

## Transparency declarations

None of the authors have a conflict of interest. Helio S. Sader, John H. Kimbrough, Marisa L. Winkler, Krisztina M. Papp-Wallace, Mariana Castanheira and Rodrigo E. Mendes are employees of Element Iowa City (JMI Laboratories), which was paid consultant to Pfizer in connection with the development of manuscript. Element Iowa City (JMI Laboratories) was contracted to perform services in 2022–2024 for AbbVie, AimMax Therapeutics, Amicrobe, Inc., Appili Therapeutics, Armata Pharmaceuticals, Astellas Pharma, Inc., Basilea Pharmaceutica AG, Biosergen AB, Bugworks, Cerba Research NV, Cidara Therapeutics, Cipla USA Inc., ContraFect Corporation, CorMedix Inc., Crestone, Inc., Curza Global, LLC, Diamond V, Discuva Ltd, Entasis Therapeutics, Enveda Biosciences, Evopoint Biosciences, Fedora Pharmaceuticals, Fox Chase Chemical Diversity Center, Genentech, Gilead Sciences, Inc., GSK plc, Iterum Therapeutics plc, Janssen Biopharma, Johnson & Johnson, Kaleido Biosciences, LifeMine Therapeutics, Medpace, Inc., Lysovant Sciences, Inc., Meiji Seika Pharma, Melinta Therapeutics, Menarini Group, Merck & Co., MicuRx Pharmaceutical Inc., Mundipharma International Ltd, Mutabilis, Nabriva Therapeutics, National Cancer Institute, National Institutes of Health, Ohio State University, Omnix Medical Ltd, Paratek Pharmaceuticals, PolyPid Ltd, PPD, Prokaryotics, Inc., Pulmocide Ltd, Qpex Biopharma, Revagenix, Roche Holding AG, Roivant Sciences, Scynexis, Inc., Shionogi & Co., Ltd, Sinovent Pharmaceuticals, Inc., Spero Therapeutics, Sumitovant Biopharma, Inc., TenNor Therapeutics, U.S. Food and Drug Administration, VenatoRx Pharmaceuticals, Washington University, Watershed Medical, LLC, Wockhardt and Zoetis, Inc.
